# Gender differences in trachomatous scarring prevalence in a formerly trachoma hyperendemic district in Tanzania

**DOI:** 10.1371/journal.pntd.0011861

**Published:** 2024-01-26

**Authors:** Meraf A. Wolle, Beatriz E. Muñoz, Glory Mgboji, Fahd Naufal, Michael Saheb Kashaf, Harran Mkocha, Sheila K. West

**Affiliations:** 1 Dana Center for Preventive Ophthalmology, Wilmer Eye Institute, Johns Hopkins University, Baltimore, Maryland, United States of America; 2 Kongwa Trachoma Project, Kongwa, Tanzania; Mohammed Bin Rashid University of Medicine and Health Sciences, UNITED ARAB EMIRATES

## Abstract

**Background:**

Trachoma is a chronic conjunctivitis caused by the bacterium *Chlamydia trachomatis*. Repeated infections lead to trachomatous conjunctival scarring which can progress to potentially blinding trachomatous trichiasis (TT). In trachoma hyperendemic conditions, women compared to men have an increased risk of scarring and TT, which can progress to blinding corneal opacification. This study determined if there were gender differences in scarring prevalence and severity when trachoma prevalence approaches elimination, in a formerly trachoma hyperendemic region.

**Methodology/Principal findings:**

A cross-sectional prevalence study was conducted amongst adults age 15 years and older in Kongwa district, Tanzania in 2019. 3168 persons over age 15 years agreed to be examined and had at least one eye with a gradable image. Ocular photographs were graded for scarring according to a published four-step severity scale. Overall, about half of all study participants had scarring. However, more females (52.3%) had any scarring compared to males (47.2%), OR = 1.22 (95% CI = 1.05–1.43). For every year increase in age, there was a 6.5% increase in the odds of having more severe scarring (95% CI: 5.8%, 7.2%). Women were more likely than men to have severe scarring, OR 2.36 (95% CI: 1.84–3.02). Residence in a community with TF≥10% was associated with a 1.6-fold increased odds of any scarring.

**Conclusions/Significance:**

Overall scarring prevalence and more severe scarring prevalence was higher in females compared to males, even adjusting for age and community TF prevalence. The data suggest that processes occur that lead to women preferentially progressing towards more severe scarring compared to men.

## Author summary

Trachoma is a chronic conjunctivitis caused by the bacterium *Chlamydia trachomatis*. Repeated infections lead to trachomatous conjunctival scarring (TS) which can progress to potentially blinding trachomatous trichiasis (TT). In trachoma hyperendemic conditions, women have an increased risk, scarring and TT. This study determined if there were gender differences in scarring prevalence and severity when trachoma prevalence approaches elimination, in a formerly trachoma hyperendemic region.

Overall, about half of all study participants had scarring. Scarring and severity of scarring increased with age. However, compared to males, more females had any scarring and they had more severe scarring.

Scarring prevalence and more severe scarring prevalence was higher in communities with higher active trachoma prevalence. Adjusting for age and community active trachoma prevalence, women still had more scarring and severe scarring compared to men. The data suggest that processes occur that lead to women preferentially progressing towards more severe scarring compared to men. Gaining a better understanding of what may be causing this increased progression, could lead to the identification of a potential modifiable risk factor.

## Introduction

Trachoma is a chronic conjunctivitis cause by the bacterium *Chlamydia trachomatis* [1]. 125 million people are at risk of blindness, and 1.9 million adults are visually impaired or irreversibly blind, from late-stage trachoma sequelae. Active trachoma, seen in children, is characterized by conjunctival inflammation which presents as trachomatous inflammation—follicular (TF) and trachomatous inflammation—intense (TI). Repeat bouts of active trachoma in children leads to trachomatous conjunctival scarring in young adults. Scarring can progress further to the in-turning of the eyelid (entropion) as well as the in-turning of eyelashes (trachomatous trichiasis, TT). TT, if not corrected, can result in the repeated breakdown of the corneal surface, placing individuals at high risk of irreversible visual loss from corneal opacification [1–3].

Active trachoma prevalence globally has decreased as a result of concerted public health efforts. However, research has shown that scarring due to trachoma can progress despite low TF rates and without continued exposure to re-infection, likely due to ongoing inflammatory processes [2,4–6]. Studies from Sub-Saharan Africa have shown that women have an increased risk of the early stages of trachoma, infection and inflammation, in addition to as much as a fourfold increased risk of the later potentially blinding stages of trachoma, scarring and TT, as compared to men; women are also more likely to develop the blinding sequelae, corneal opacity [7–11]. These studies were conducted under trachoma hyperendemic conditions, and except for one study, severity of scarring was not assessed. Once a district achieves low prevalence of TF, it is not clear if this increased risk of scarring in women remains. Furthermore, it is not clear whether the increased risk of potentially blinding sequalae arise from women simply developing more scarring overall (i.e., women have excess scarring in all grades of severity) or if there is a component due to women preferentially progressing to more severe scarring at higher rates.

We conducted a cross-sectional prevalence study in a formerly trachoma hyperendemic region, now with a TF prevalence of 7%, to evaluate whether there are gender differences in scarring prevalence and severity.

## Methods

### Ethics statement

This study was approved by both the Johns Hopkins School of Medicine Institutional Review Board and the National Institute for Medical Research in Tanzania. Written consent was obtained from study participants either in Swahili or in their local language.

### Population

A cross-sectional prevalence study was conducted in Kongwa district, Tanzania in 2019. 50 communities were randomly selected followed by 50 households in each community, as described in a previous publication [12]. All adults age 15 years and older registered during the census who were living in the house at the time of the ocular exam were eligible for this study.

Kongwa district is a formerly trachoma hyperendemic district where TF in 1986 was 60% in those age 7 years and under. Interventions started in a few villages in 1991, but coverage was very incomplete. Full coverage with MDA occurred starting in 2008, when TF prevalence was 31%. In 2016, the estimated prevalence of TF had fallen to 5% [13,14]. At the start of this study the TF prevalence was 7% [12].

### Data collection

Data obtained on participants included age, gender, community TF prevalence in children ages 1–9 years, and photographs of the everted upper eyelid.

### Examination of scarring

**Fig 1:**
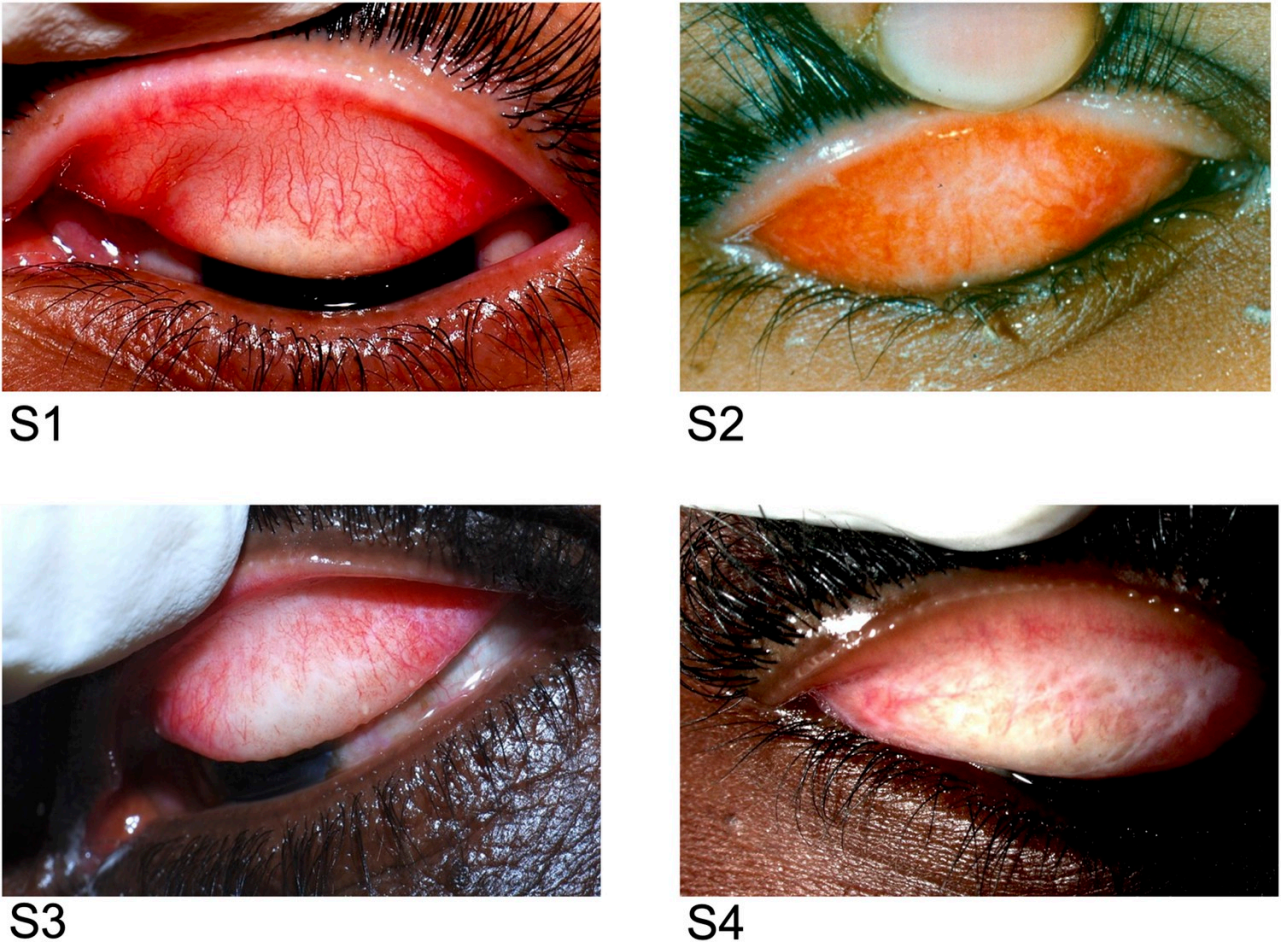
Examples of grades of trachomatous scarring from S1 to S4. Original source: Wolle MA, Muñoz B, Mkocha H, West SK. Age, sex, and cohort effects in a longitudinal study of trachomatous scarring. Invest Ophthalmol Vis Sci. 2009 Feb;50 [2]:592–6 [15]. Copyright is held by ARVO.

Tarsal photographs were taken with a handheld Nikon D-series camera (D-40) with a 105mmf/2·8D AF Macro Nikkor Autofocus Lens (in manual setting) by a trained team member. [15]. The tarsal photographs were graded for scarring according to a previously described four-step severity scale based on photographs [15]. Briefly, there were four trained graders all of whom underwent a multi-day training and achieved a kappa of > 0.7 with our senior graders prior to starting grading. Each image had two graders and any disagreements were openly adjudicated with the senior expert. The grading scale used includes the following severity levels: S0 (none), S1 (minimal), S2 (moderate), S3 (severe), and S4 (very severe). See Fig 1.

### Statistical analysis

The analysis was done at the person level; for each participant the more severely affected eye was selected. Bivariate analyses examined the effect of age, gender, and community TF prevalence on the presence and severity of scarring. For the multivariate analysis, we attempted an ordinal model, however the proportional log-odds assumption did not hold. Therefore, we analyzed the odds of any scarring compared to no scarring (S0), and the odds of severe scarring (grades 3 and 4) compared to non-severe and no scarring (grades S0-S2). Logistic regression models were used to examine the association between the age, gender, and scarring severity. The generalized estimated equation (GEE) approach was used to correct the standard errors to account for the within-village correlation. The 10-year age specific incidence of scar grades S3 or S4 was estimated using the following formula I_x_ = (P_x+10_ –P_x_)/(1 –P_x_), where I_x_ is the incidence rate at age X, P_x_ is the prevalence at age X and P_x+10_ is the prevalence at age X+10 [16,17]. All analyses were caried out using SAS 9.04.01 M6 software.

## Results

**Fig 2:**
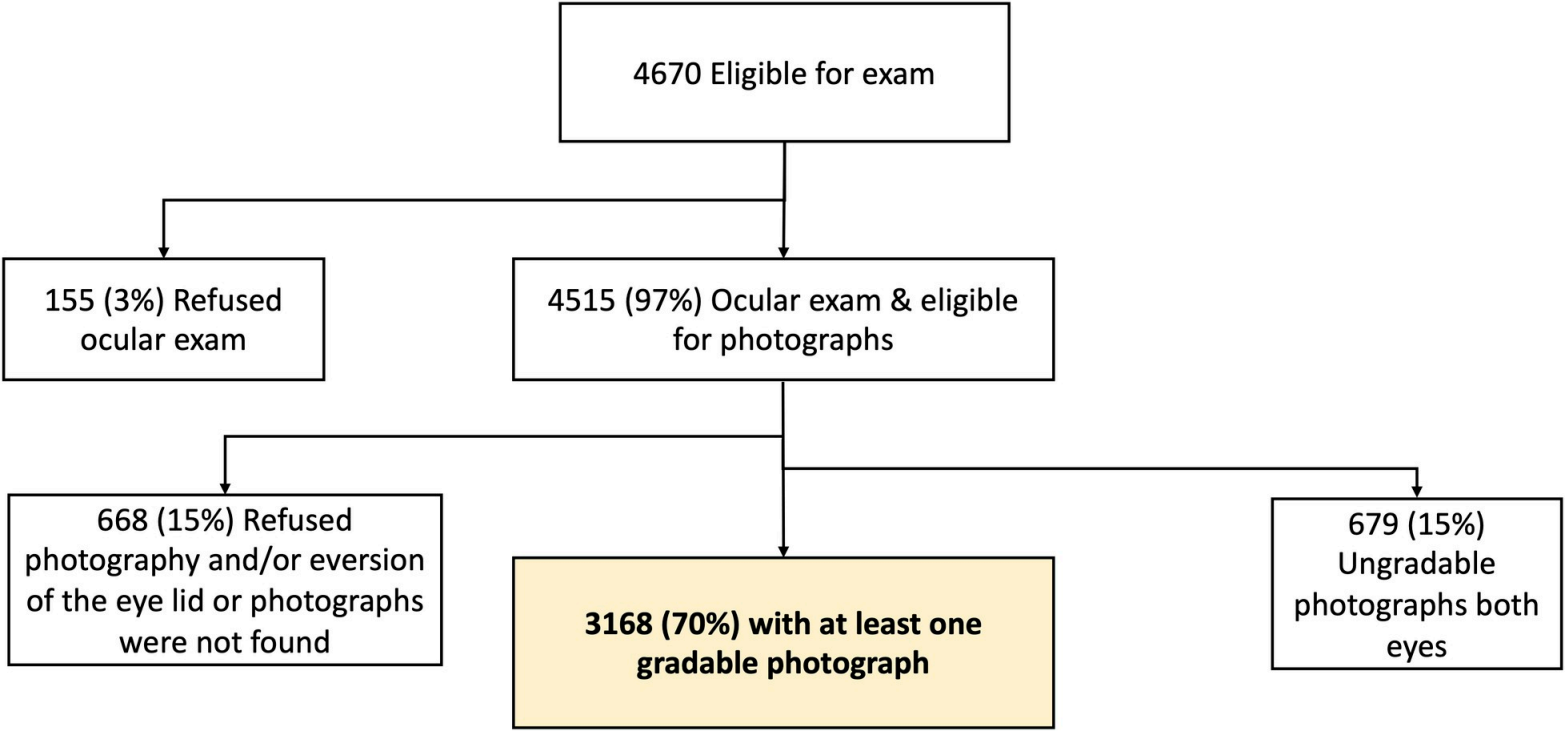
Schema of study participation.

A total of 4670 persons over age 15 years were identified from a census and eligible for this study. Of the 4670, 155 (3%) refused the exam. Of the 4515 who participated, 3168 participants (70%) had at least one eye with gradable images. Fig 2 shows participant inclusion.

The 3168 participants’ mean age was 36.1 years (SD 17.4) and 60.1% were female. There were differences in the demographic characteristics of study participants compared to those not included in the study who had an ocular exam but no graded images (Table 1). Those not included in the study were older and slightly more likely to be female and to come from communities where the TF prevalence was still >10%.

**Table 1:** Demographic characteristics of those included in the study compared to those not included.

Characteristic	Included N = 3168	Excluded N = 1347	p-value
**Age in years (Mean (SD))**	36.1 (17.4)	42.6 (18.2)	<0.0001
**Age category (n (%))**			
15–19	622 (19.6)	128 (9.5)	<0.0001
20–29	770 (24.3)	250 (18.6)	
30–39	618 (19.5)	268 (19.1)	
40–49	499 (15.8)	269 (20.0)	
50–59	289 (9.1)	176 (13.1)	
60–69	190 (6.0)	112 (8.3)	
70+	181 (5.7)	143 (10.6)	
**Gender (n (%))**			
Male	1265 (39.9)	489 (36.3)	0.02
Female	1903 (60.1)	858 (63.7)	
**Community TF prevalence (n(%))**			
<5%	1277 (40.3)	489 (36.3)	0.004
5%-<10%	962 (30.4)	399 (29.6)	
≥10%	929 (29.3)	459 (34.1)	

**Fig 3:**
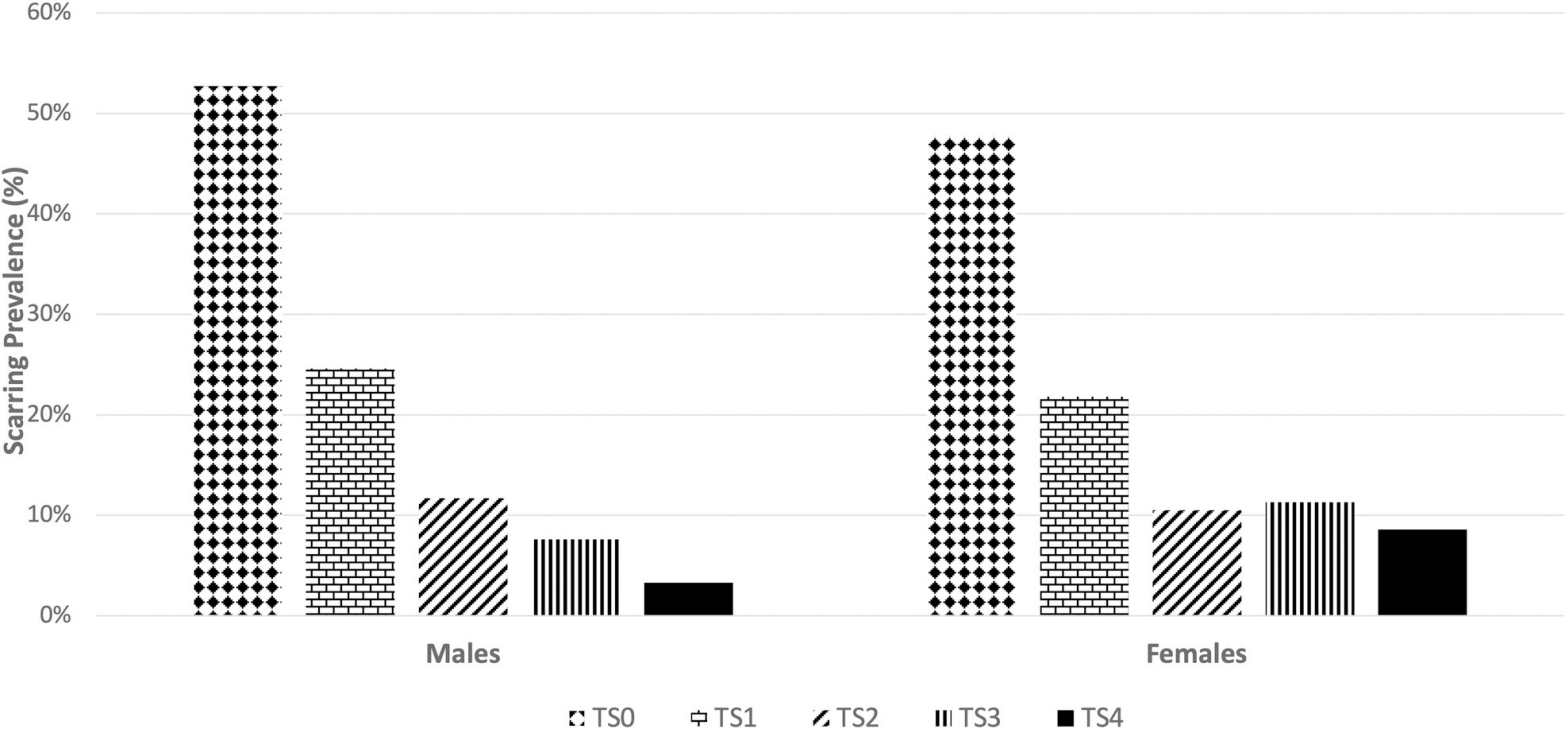
Scarring severity distribution by gender.

Overall, about half of all study participants had scarring. However, more females (52.3%) had any scarring compared to males (47.2%), OR = 1.22 (95% CI = 1.05–1.43). Males and females had similar prevalence of minimal (S1) and moderate (S2) scarring; females had an increased prevalence of more severe scarring (S3 and S4 scars) (Fig 3).

**Fig 4:**
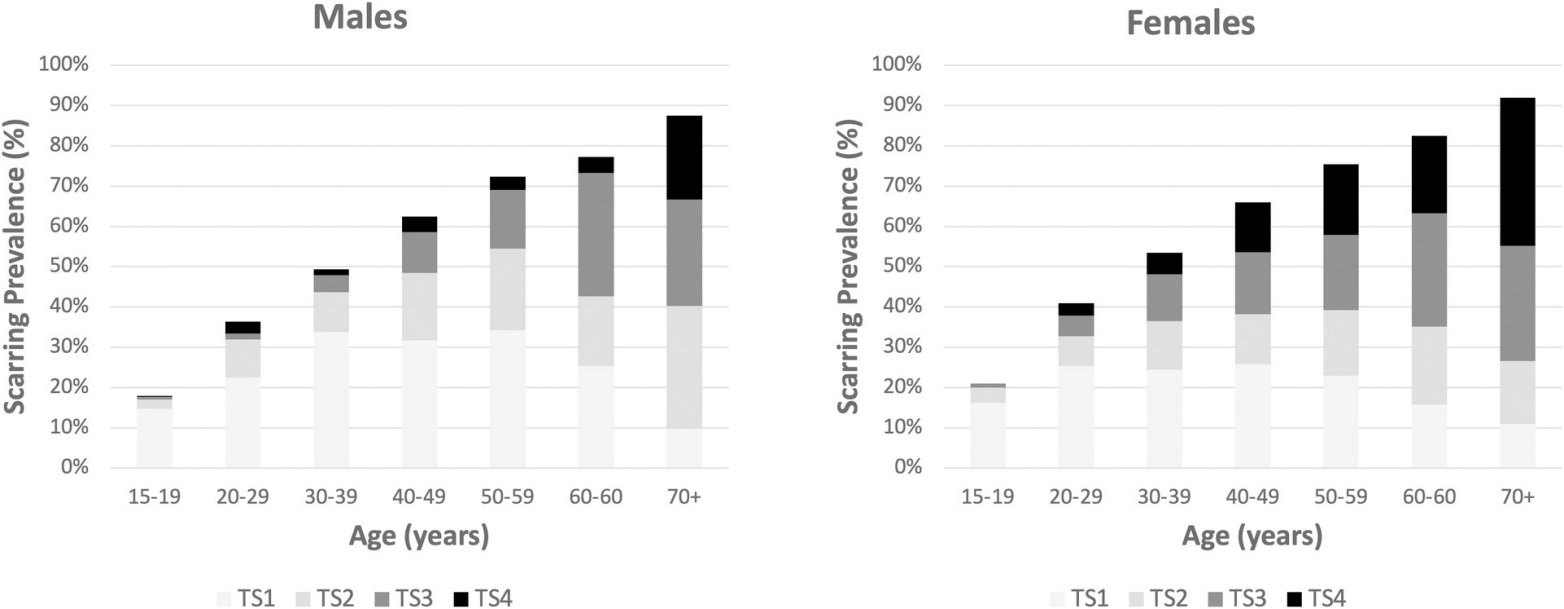
Scarring severity distribution by age group and gender.

Scarring severity increased by age within each gender (Fig 4). Age was associated with more severe scarring; for every year increase in age, there was a 6.5% increase in the odds of having more severe scarring (95% CI: 5.8%, 7.2%). Adjusting for age, women were more likely than men to have severe scarring, OR 2.36 (95% CI: 1.84–3.02).

**Fig 5:**
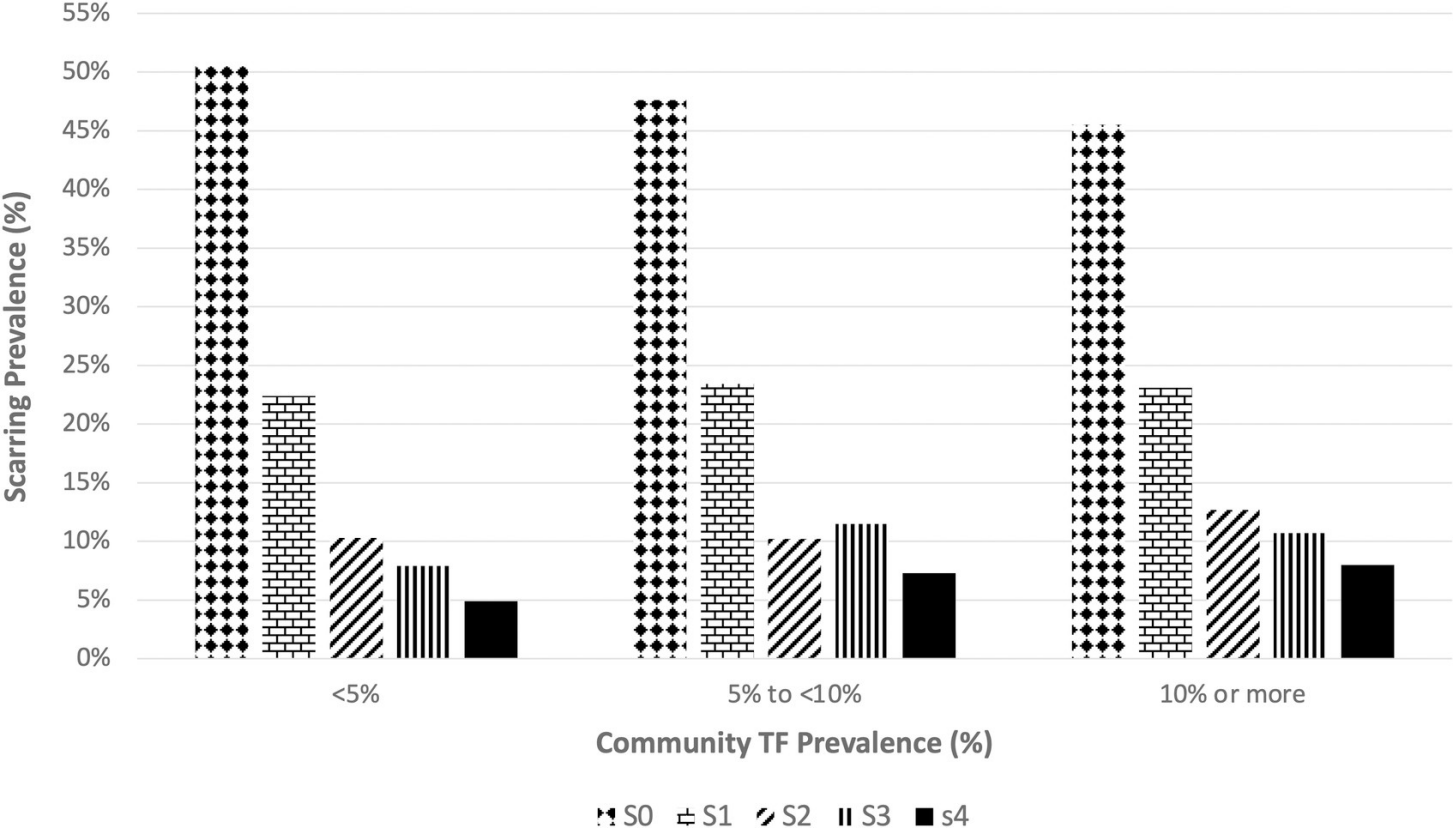
Scarring prevalence by Community TF prevalence measured in 1–9 year old children.

The prevalence of TF, as measured by the survey in children ages 1–9 years [12], was also related to scarring and the severity of scarring (Fig 5).

A multivariate model for any scarring shows independent contributions of increasing age (OR 1.06, 95% CI: 1.057–1.069), female sex (OR 1.22, 95% CI: 1.05–1.32), and residence in a community with estimated TF prevalence ≥10% (Table 2). The increased odds of scarring with residence in communities with TF prevalence between 5 and 9.9% was not statistically significant. A multivariate model for severe scarring showed the same independent risk factors found for any scarring but also found a significant odds ratio for community TF prevalence from 5 to 9.9% (OR 1.61, 95% CI: 1.10–2.37) as well as TF prevalence greater than or equal to 10% (OR 1.87, 95% CI: 1.23–2.85) (Table 3).

**Table 2:** Multivariate model of the odds of any scarring.

Variable	Odds Ratio	95% CI	P value
Age (per year)	1.063	(1.057–1.069)	<0.0001
Female vs male	1.22	(1.05–1.32)	<0.011
TF 5% to <10%	1.36	(0.92–2.01)	0.13
TF ≥10%	1.55	(1.11–2.17)	0.0105

* Models account for within community correlation

**Table 3:** Multivariate model of the odds of severe scarring.

Variable	Odds Ratio	95% CI	P value
Age (per year)	1.065	(1.058–1.072)	<0.0001
Female vs male	2.36	(1.84–3.02)	<0.0001
TF 5 to <10%	1.61	(1.10–2.37)	0.015
TF ≥10%	1.87	(1.23–2.85)	0.0035

**Fig 6:**
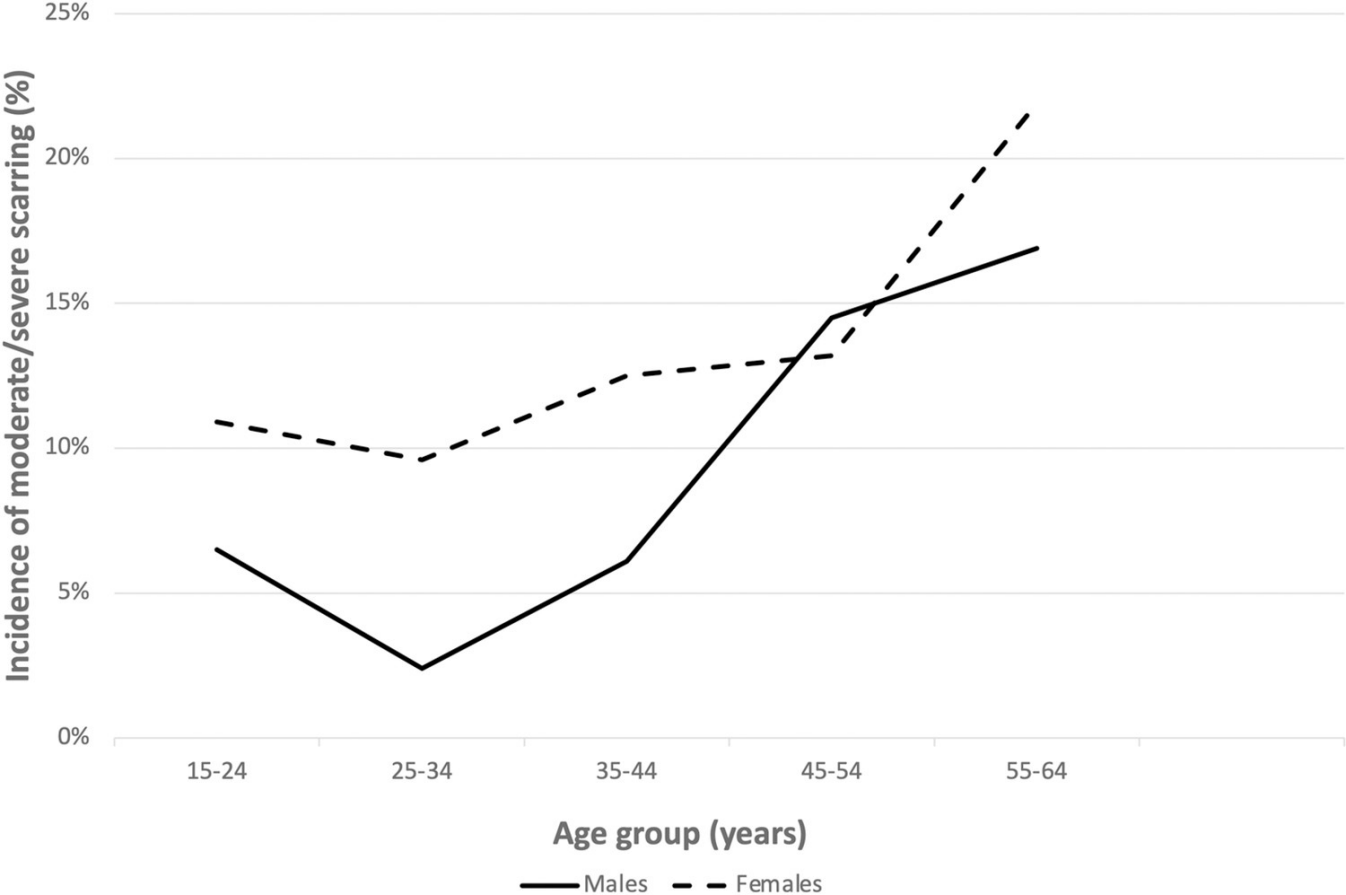
Estimated 10-year incidence of moderate/severe scarring by age group and gender.

The cross-sectional data on age and gender and scarring severity was used to create estimates of the 10-year incidence of severe/very severe scarring. The graph shows that the incidence of severe scarring at each ten-year age band was greater for females compared to males, except at the age group 45–54 where they were similar. (Fig 6).

## Discussion

In this cross-sectional study of trachoma prevalence in a formerly trachoma hyperendemic district with a low overall rate of TF (7%), overall scarring prevalence and more severe scarring prevalence increased with age and in females compared to males.

Our study shows that the prevalence of any scarring, as well as the prevalence of more severe scarring, increases with age. This finding is similar to those in other prevalence and longitudinal surveys conducted in both low and high TF prevalence settings [7,15,18,19]. It is also consistent with the biology of trachoma; multiple episodes of infection are needed to develop scarring, as a result, scarring usually starts in young adults and as time goes by, more and more adults develop scarring [2]. It also takes time for new scarring to progress from mild to severe, thus an accumulation of more severe scarring is seen as age increases [2]. We have already reported that the incidence of new scars appears to decline once TF decreases [16]. Although the prevalence appears to be slightly lower in the age group 15–19 years, it is likely too soon to see the effects of low TF prevalence in this district on incident scarring. Further follow up may chart the impact with time on scarring.

Our study also shows that severe scars are more likely in communities with TF prevalence of 5% or more, and that this likelihood increases in communities with a TF of 10% or more. A likely explanation is that these communities that still have active trachoma in a district where trachoma rates were 7% represent communities that were very high in the past [20] and are more slowly declining. Individuals living in these communities have had more intense TF transmission in the past leading to early onset of scarring and early progression of scarring. This assumes this current high TF rate is also indicative of prior high TF rates, an assumption which is reasonable given the way TF declines with MDA [21,22]. Research has shown that scarring can progress even after low *Ct* infection and active trachoma levels, likely due to ongoing inflammatory processes [2,4–6,18,23,24] so at this point we do not expect that the trachoma rates are driving progression, but are rather indicative of past high exposure.

Women had an increased prevalence of any scars as compared to men. Women are thought to have more scarring compared to men due to their exposure to children who are the reservoir of infection in their communities. Under hyperendemic conditions, it would be difficult to find women who were not exposed, and indeed the older women in this study likely did have greater exposure to children and thus infection compared to men. However, as TF declines, we have observed a decline in incident scarring in women [18]; it is still related to TF prevalence, but as TF declines, so does onset of scarring. However, progression of scarring does not appear to change in the face of decline [19]. Previous studies have reported increased scarring in females compared to males, however, these studies were conducted under high TF conditions and except for one study, none detailed the severity of scarring [8,10,11,20,25]. Interestingly, the increased scarring prevalence we are seeing is no longer a four-fold increased risk of scarring as was seen under hyperendemic conditions suggesting the differential between men and women is declining as the TF prevalence declines [10].

Women also had a higher risk of severe scarring in all age groups, and the age specific incidence of severe scarring model suggested that women progressed to severe scarring at younger ages compared to men. It also suggests that the reason the cross-sectional prevalence of mild scarring is similar in men and women is that men do not progress as rapidly as women do but stay in lower severities of scarring, while women progress out of stage 1 and 2 scarring. One potential explanation for women having higher rates of scarring progression is that women are more likely to have an abnormal persistent inflammatory response to trachoma infection. This is consistent with underlying biological factors that lead to women having a higher prevalence of certain autoimmune conditions which result from the body’s abnormal inflammatory response to a presumed antigen [26]. Another potential explanation for women having higher rates of scarring progression could be due to other concurrent bacterial co-infections contributing to conjunctival scarring progression, although it is not clear why women would have more infections compared to men [17]. A study by Cevallos et al looked at individuals with trichiasis and found that women were more likely to be colonized by non-chlamydial bacteria compared to men [27], but patients with trichiasis have severe ocular surface disease and it is unclear if the colonization led to trichiasis or was the result of trichiasis. The studies on non-chlamydial ocular infections and scarring are mixed [28–30].

There are limitations to the study. One is the loss of participants due to refusal to have images, and poor-quality images. Those not included were older, female, and came from communities with higher TF prevalence. Thus, the exclusion of these individuals may have resulted in an underestimation of scarring prevalence, particularly in women. The relative absence of men in the census and thus in the study population, has been seen in other studies. The small numbers make estimates of scarring and particularly severe scarring in men more unstable in the older ages. If those males with more severe scarring tended to be at home, then we could have overestimated severe scarring in males, and this could explain the large jump in rates of S4 in the male population age 70 and older. We also must be cautious in interpreting the model of incident S3 and S4 scarring. We chose a model that estimates incidence from cross sectional data, and that has been used by others [16]. We do note the model, derived from age and gender prevalence estimates, assumes a stable risk over time, without a cohort effect, and that is not the case when these communities have been under persistent efforts to eliminate trachoma including antibiotic pressure and interventions to improve facial cleanliness and environmental hygiene for the last ten years. The model also assumes that the distribution of scarring severity within a grade are similar by gender, and that may not be the case. For example, if females are more likely to be at the higher end of the S1 and S2 categories compared to males, they would be more likely over the same time-period to progress to more severe scarring. Finally, as discussed above, there is missing data not at random, and that can influence the assumptions of the representativeness of the prevalence at each age. The strengths of this study are the large sample size and the use of a valid, reliable method for grading scarring severity.

In conclusion, the data suggest that in this low TF prevalence district, scarring is still somewhat greater in women, but no longer at a four-fold increased risk. Moreover, the data suggest that processes occur that lead to women preferentially progressing towards more severe scarring compared to men at odds ratios similar to that seen in high TF prevalence districts. Gaining a better understanding of what may be causing this increased progressioncould lead to the identification of a potential modifiable risk factor.

## Supporting information

S1 File STROBE checklist. (PDF)

S2 File Supporting data. (XLSX)
