# Supplementary material for: Gender differences in trachomatous scarring prevalence in a formerly trachoma hyperendemic district in Tanzania
Source: PLoS Negl Trop Dis. 2024 Jan 26;18(1):e0011861. doi: 10.1371/journal.pntd.0011861 (PMC10817155; doi:10.1371/journal.pntd.0011861)
Supplement: S1 File — (PDF) [file pntd.0011861.s001.pdf]

Section/Topic	Item No.	Recommendation	Reported in Paper
**Title and abstract**	1	(*a*) Indicate the study's design with a commonly used term in the title or the abstract	Page 1, Line 1
		(*b*) Provide in the abstract an informative and balanced summary of what was done and what was found	Page 2-3, Line 18-47
**Introduction**			
Background/rationale	2	Explain the scientific background and rationale for the investigation being reported	Page 5-6, Line 72-103
Objectives	3	State specific objectives, including any prespecified hypotheses	Page 6, Line 101-103
**Methods**			
Study design	4	Present key elements of study design early in the paper	Page 6, Line 114-118
Setting	5	Describe the setting, locations, and relevant dates, including periods of recruitment, exposure, follow-up, and data collection	Page 6, Line 114-118
Participants	6	(*a*) Give the eligibility criteria, and the sources and methods of selection of participants. Describe methods of follow-up	Page 6, Line 114-118
		(*b*) For matched studies, give matching criteria and number of exposed and unexposed	N/A
Variables	7	Clearly define all outcomes, exposures, predictors, potential confounders, and effect modifiers. Give diagnostic criteria, if applicable	Page 6-7, Line 130-138
Data sources/ measurement	8*	For each variable of interest, give sources of data and details of methods of assessment (measurement). Describe comparability of assessment methods if there is more than one group	Page 6-7, Line 130-138
Bias	9	Describe any efforts to address potential sources of bias	Page 8, Line 146-158
Study size	10	Explain how the study size was arrived at	Page 6, Line 114-118
Quantitative variables	11	Explain how quantitative variables were handled in the analyses. If applicable, describe which groupings were chosen and why	Page 8, Line 146-158
Statistical methods	12	(*a*) Describe all statistical methods, including those used to control for confounding	Page 8, Line 146-158
		(*b*) Describe any methods used to examine subgroups and interactions	N/A
		(*c*) Explain how missing data were addressed	N/A
		(*d*) If applicable, explain how loss to follow-up was addressed	N/A
		(*e*) Describe any sensitivity analyses	N/A
**Results**			
Participants	13*	(a) Report numbers of individuals at each stage of study—eg numbers potentially eligible, examined for eligibility, confirmed eligible, included in the study, completing follow-up, and analysed	Page 9, Line 162-165
		(b) Give reasons for non-participation at each stage	Page 9, Line 162-165
		(c) Consider use of a flow diagram	Figure 2
Descriptive data	14*	(a) Give characteristics of study participants (eg demographic, clinical, social) and information on exposures and potential confounders	Page 10, Line 170-174, Table 1
		(b) Indicate number of participants with missing data for each variable of interest	N/A
		(c) Summarise follow-up time (eg, average and total amount)	N/A
Outcome data	15*	Report numbers of outcome events or summary measures over time	Page 10, Line 180-183, Figure 3
Main results	16	(*a*) Give unadjusted estimates and, if applicable, confounder-adjusted estimates and their precision (eg, 95% confidence interval). Make clear which confounders were adjusted for and why they were included	Page 11, Line 188-211, Figure 4-5, Table 2
		(*b*) Report category boundaries when continuous variables were categorized	Page 11, Line 188-211, Figure 4-5, Table 2

		(*c*) If relevant, consider translating estimates of relative risk into absolute risk for a meaningful time period	N/A
Other analyses	17	Report other analyses done—eg analyses of subgroups and interactions, and sensitivity analyses	Page 14. Line 221-225, Figure 6.
**Discussion**			
Key results	18	Summarise key results with reference to study objectives	Page 15, line 233-236
Limitations	19	Discuss limitations of the study, taking into account sources of potential bias or imprecision. Discuss both direction and magnitude of any potential bias	Page 18-19, 298-319
Interpretation	20	Give a cautious overall interpretation of results considering objectives, limitations, multiplicity of analyses, results from similar studies, and other relevant evidence	Page 19, Line 321-327
Generalisability	21	Discuss the generalisability (external validity) of the study results	Page 19, Line 321-327
**Other information**			
Funding	22	Give the source of funding and the role of the funders for the present study and, if applicable, for the original study on which the present article is based	In submission information
